# Tough Call: Challenges to Assessing Cancer Effects of Mobile Phone Use

**Published:** 2009-03

**Authors:** M. Nathaniel Mead

Mobile phone use worldwide has exploded in the past decade, with many countries fast approaching a usage prevalence of 100%. Even as mobile phone use grows exponentially, questions remain regarding the health impact of frequent exposure to the electromagnetic fields (EMFs) associated with mobile phone use. A review of 33 peer-reviewed epidemiologic studies suggests that a number of study design issues may result in an underestimate of the relative risk of brain tumors among mobile phone users **[*EHP* 117:316–324; Kundi]**.

Recall bias is a widely cited concern that could lead to biased risk estimates in case–control studies of ipsilateral exposure (i.e., tumors occurred on the same side of the head where the phone was usually held); the review author notes that cancer patients may tend to either attribute their disease to their mobile phone use or to dismiss a relationship between the two. But ipsilateral risks also carry greater biologic plausibility, since one 2008 study showed that nearly 99% of the total electromagnetic energy deposited in the brain is absorbed at the side of the head where the phone is held during calls. According to the author’s analysis, more than half of mobile phone users among cases and none among controls would have to incorrectly identify which ear they usually hold their phone to in order to nullify the observed increased risk.

Another source of potential bias concerns the comparison groups used. In the widely cited Interphone study, a case–control study spanning 13 countries, the unexposed group included people who used cordless phones. However, according to the author, cordless and mobile phones users receive about the same EMF exposure, and cordless phones are generally used for longer periods of time than mobile phones. This may help explain why Interphone has consistently reported either no effect or even a protective effect of mobile phone use.

Finally, methods of data acquisition, which have differed substantially between Interphone and other studies, may also introduce bias. Memory performance may be altered in patients with aggressive gliomas, malignant brain tumors that have been associated with mobile phone use in some studies. The author also suggests that exposure assessment may be biased if conducted by phone interviews (as in the Interphone study) compared with the mailed questionnaire method.

According to the review author, results of the research to date suggest an association between mobile phone use and glioma risk that falls in the range of magnitude delineated for passive smoking and lung cancer. Confidence in a causal relationship is bolstered by two key findings: longer latencies are associated with higher risk estimates, and living in a rural area—where mobile phones typically radiate at higher intensities—also is associated with elevated risk. Even a modest cancer risk could have major public health implications because of the vast number of mobile phone users. On the other hand, as this review points out, the individual risk perspective is less dramatic: in industrialized countries, the prevailing life-time brain tumor risk is 4–8 per 1,000, and thus individual risk is still low if mobile phone use increases the risk even 50% to 6–12 per 1,000.

## Figures and Tables

**Figure:**
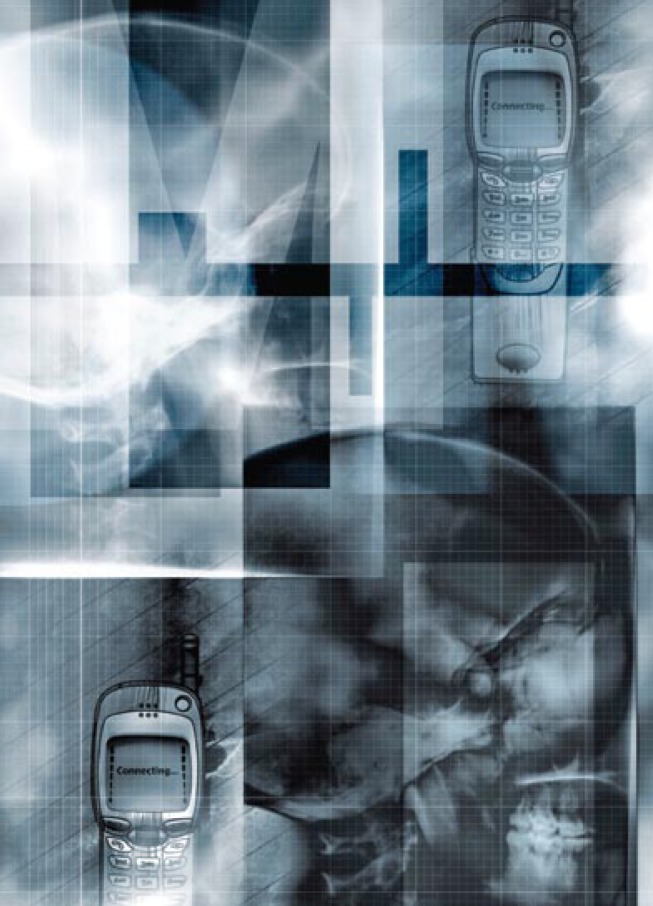
A review of mobile phone use and cancer reveals areas where study design can be strengthened.

